# Autoimmune and Metabolic Diseases and the Risk of Early-Onset Colorectal Cancer, a Nationwide Nested Case–Control Study

**DOI:** 10.3390/cancers15030688

**Published:** 2023-01-22

**Authors:** Erik Lundqvist, Ida Hed Myrberg, Sol Erika Boman, Deborah Saraste, Caroline E. Weibull, Kalle Landerholm, Staffan Haapaniemi, Anna Martling, Pär Myrelid, Caroline Nordenvall

**Affiliations:** 1 Department of Biomedical and Clinical Sciences, Linköping University, 58183 Linköping, Sweden; 2 Department of Surgery, Vrinnevi Hospital, 60379 Norrköping, Sweden; 3 Department of Medicine, Division of Clinical Epidemiology, Karolinska University Hospital, Karolinska Institutet, 17177 Stockholm, Sweden; 4 Department of Surgery, Södersjukhuset, 11883 Stockholm, Sweden; 5 Department of Molecular Medicine and Surgery, Karolinska Institutet, Division of Coloproctology, Center for Digestive Diseases, Karolinska University Hospital, 17177 Stockholm, Sweden; 6 Department of Surgery, Ryhov County Hospital, 55305 Jönköping, Sweden; 7 Department of Surgery, Linköping University Hospital, 58183 Linköping, Sweden

**Keywords:** early-onset colorectal cancer, risk factors, autoimmune disease, inflammatory bowel disease, metabolic disease, hypertension, diabetes mellitus, hyperlipidemia, obesity

## Abstract

**Simple Summary:**

Early onset of colorectal cancer (EOCRC) is increasing in developed countries. The aim was to investigate autoimmune and metabolic conditions as risk factors for EOCRC. We investigated preexisting autoimmune and metabolic diagnoses of 2626 EOCRC patients in Sweden, diagnosed in 2007–2016, together with 15,756 controls matched for birth year, sex, and county. Comorbid diagnoses were collected from the National Patient Register. A history of metabolic disease nearly doubled the incidence of EOCRC, and presence of inflammatory bowel disease (IBD) was associated with a sixfold increased incidence of EOCRC. Patients with both IBD and metabolic disease had a lower incidence of EOCRC compared with IBD patients without metabolic condition. Non-IBD autoimmune disease was not associated with an increased incidence of EOCRC. IBD and metabolic disease are risk factors for EOCRC and should be considered in screening guidelines.

**Abstract:**

Incidence of early-onset (<50 years) colorectal cancer (EOCRC) is increasing in developed countries. The aim was to investigate autoimmune and metabolic conditions as risk factors for EOCRC. In a nationwide nested case–control study, we included all EOCRC cases in Sweden diagnosed during 2007–2016, together with controls, matched for birth year, sex, and county. Information on exposure of autoimmune or metabolic disease was collected from the National Patient Register and Prescribed Drugs Registry. Hazard ratios (HR) as measures of the association between EOCRC and the exposures were estimated using conditional logistic regression. In total, 2626 EOCRC patients and 15,756 controls were included. A history of metabolic disease nearly doubled the incidence hazard of EOCRC (HR 1.82, 95% CI 1.66–1.99). A sixfold increased incidence hazard of EOCRC (HR 5.98, 95% CI 4.78–7.48) was seen in those with inflammatory bowel disease (IBD), but the risk increment decreased in presence of concomitant metabolic disease (HR 3.65, 95% CI 2.57–5.19). Non-IBD autoimmune disease was not statistically significantly associated with EOCRC. IBD and metabolic disease are risk factors for EOCRC and should be considered in screening guidelines.

## 1. Introduction

Colorectal cancer (CRC) is the third most common malignancy, both globally and in Sweden, and the second leading cause of cancer-related death [1,2]. Age-standardized incidence of CRC has been slowly decreasing since the 1990s owing to a more widespread use of screening tests such as fecal occult blood test and colonoscopy, which can identify precursor lesions [3]. On the contrary, several large population-based studies have reported an increased incidence of early-onset CRC (EOCRC), often defined as age <50 years at diagnosis [3,4,5]. It is estimated that, by 2030, 10% of colon cancers and 22% of rectal cancers will occur in patients under 50 years of age in the United States, compared to the current 4% and 9%, respectively [6]. In a Swedish study, the incidence of both colon and rectal cancer increased over time, especially among women [5]. Compared to late-onset CRC (LOCRC), EOCRC has a predilection for the left-sided colon and rectum and is associated with a more advanced stage at diagnosis, as well as more adverse clinicopathological findings [7]. Although EOCRC patients receive more intense oncological treatment, the survival benefit of this is unclear [7,8].

The risk factors for CRC development at any age are well studied [9]. In summary, genetic predisposition plays an important role, as does increasing age. Men are more prone to rectal cancer compared to women, while colon cancer has a relatively equal distribution between men and women [10]. Lifestyle factors such as physical inactivity, obesity, diabetes mellitus type 2 (T2DM), smoking, and heavy alcohol consumption are thought to influence the risk of CRC. A diet with high intake of red and processed meat, low intake of fiber, fruits, and vegetables, and low intake of calcium, vitamin D, and dairy products has also been linked to increased risk of CRC [9,11,12,13].

Autoimmune diseases are associated with an overall increase in cancer incidence, and there is a global trend of increasing incidence of autoimmunity in general [14,15,16]. Inflammatory bowel disease (IBD), including both Crohn’s disease (CD) and ulcerative colitis (UC), and primary sclerosing cholangitis (PSC) have a clear association with CRC even if studies in EOCRC patients are scarce [17,18]. When it comes to other autoimmune diseases and the risk of CRC, there is conflicting evidence in patients with type 1 diabetes mellitus, hypothyroidism, psoriasis, rheumatoid arthritis, and spondylarthritis [14,19,20,21].

A common denominator for mentioned risk factors, including IBD, obesity, hyperlipidemia, and dietary factors, is chronic inflammation. Inflammation in the gastrointestinal tract is a possible catalyst to the origin of precancerous lesions [22]. Although risk factors for CRC development in general are established, there is a need to elucidate underlying factors contributing to the increased incidence of CRC in young adults. A recent meta-analysis concluded that obesity, hyperlipidemia, and alcohol consumption independently nearly double the risk of EOCRC [23]. However, the scientific evidence regarding the associations of IBD, autoimmune diseases, hypertension, and metabolic comorbidity with the risk of EOCRC is still scarce [23,24]. Screening recommendations generally do not include patients under the age of 50 [10,25], but a deeper understanding of the risk factors in EOCRC might impact future screening and surveillance recommendations.

### Objectives

In the present study, exposures to benign autoimmune and metabolic comorbidities and their effect on EOCRC incidence were analyzed in a nested case–control setting. We hypothesized that autoimmune diseases, including but not limited to IBD, and metabolic conditions are associated with an increased risk of EOCRC.

## 2. Materials and Methods

### 2.1. Data Source

Data were retrieved from the Colorectal Cancer database (CRCBaSe), a multi-linkage originating from the Swedish Colorectal Cancer Register (SCRCR) [26]. In CRCBaSe, data from the SCRCR are combined with data from national registers at the National Board of Welfare and Statistics Sweden, using the personal identification numbers unique to all Swedish residents [27]. In the present study, we linked data from SCRCR with the National Patient Register (NPR), Prescribed Drug Register (PDR), Register of Total Population, and the Longitudinal Integrated Database for Health Insurance and Labor Market Studies (LISA) [28].

The SCRCR is a national register with nearly complete coverage of patients with adenocarcinoma in the rectum since 1995 and in the colon since 2007, and it has been reported to have high validity [29]. Data in the SCRCR are prospectively registered during treatment and follow-up. For this study, clinical data on body measurements, ASA (American Society of Anesthesiologists) classification, tumor stage, and tumor location were collected. Colon tumors located from the right colon to transverse colon were classified as right-sided, and tumors from the splenic flexure to sigmoid colon were classified as left-sided. From the LISA database, data on disposable income per family unit and educational level were extracted. The highest achieved level of education was defined at the year of CRC diagnosis, while, for income, information on the year prior to CRC diagnosis was used. The NPR nationally covers diagnoses of inpatient care since 1987 and outpatient specialist care since 2001 [30]. We used the tenth version of the International Classification of Disease (ICD10) to identify the exposure of comorbid conditions in the NPR [31]. Data were collected for inpatient care 0–10 years prior to the CRC diagnosis and for outpatient visits 0–5 years prior to the CRC diagnosis. To strengthen the identification of persons exposed to metabolic conditions not already identified in the NPR, the Prescribed Drug Register (PDR) was used. This register, starting in 2005, is a nationwide register on all dispensed medications, apart from over-the-counter medications and drugs used in hospitals and nursing homes [32]. All drugs are classified according to the Anatomic Therapeutic Chemical (ATC) code. The PDR contains data on dispensed items, date of prescription and dispensing, dispensed amount, and dosage.

### 2.2. Study Design

**Figure 1:**
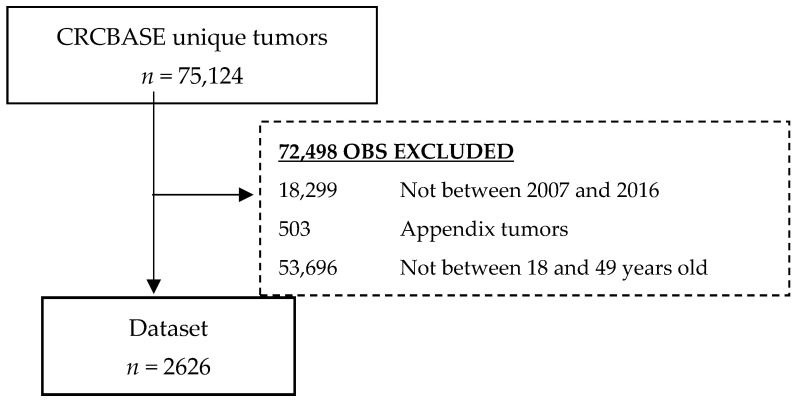
Flowchart of excluded cases.

This was a nested case–control study on the association between autoimmune and metabolic diseases and the incidence of EOCRC. Although there is no clear consensus regarding the age of early-onset colorectal cancer, EOCRC was here defined as a diagnosis of CRC before the age of 50 years, consistent with several previous articles [5,33]. Inclusion criteria were Swedish adult patients, aged ≥18 to <50 years old at diagnosis, with a first-time CRC diagnosis registered in the SCRCR between 1 January 2007 and 31 December 2016. Patients with appendix tumors were excluded (*n* = 503) (Figure 1). To each case, six controls matched for birth year, sex, and county, as well as CRC-free at time of matching, were included, resulting in a total study population of 2626 CRC patients (cases) and 15,756 controls.

### 2.3. Exposure

The exposure of interest was comorbidity in the form of either autoimmune or metabolic disease, or a combination of both. The exposure was classified according to the corresponding ICD10 or ATC codes, and diagnoses classified as autoimmune or chronic inflammatory were grouped according to their respective organ system (Supplementary Table S1). Autoimmune conditions in the gastrointestinal tract, e.g., intestinal malabsorption, primary biliary cholangitis, and autoimmune hepatitis, were grouped separately from IBD. IBD was analyzed as a group and descriptively separated into UC, CD, and IBD-unclassified (IBD-U), defined as IBD-indeterminate or records of both CD and UC in the same patient. The duration of IBD was estimated as the time from first registered diagnosis to the date of CRC diagnosis. Metabolic disease was defined as having any of the ICD10 codes for type 2 diabetes mellitus (T2DM), primary hypertension, disorders of lipoprotein metabolism and other dyslipidemias, overweight and obesity, or fatty change of liver. In order to identify as many individuals with metabolic disease as possible, we also checked the PDR for dispensed medications with ATC codes A10 for T2DM, C03, C07–C09 for primary hypertension, and C10 for hyperlipidemia (Supplementary Table S2). Dispensed medications were collected in the PDR up to 1.5 years prior to the CRC diagnosis. Exposures were also grouped into *metabolic disease*, *IBD*, and *non-IBD autoimmune disease*, where the latter included any autoimmune conditions except of IBD.

### 2.4. Statistical Analyses

Descriptive statistics were calculated as mean and standard deviation (SD) unless otherwise specified. Matched risk sets that had the same values on all matching factors were merged to gain statistical power. Hazard ratios (HRs) and corresponding 95% confidence intervals (CIs) for CRC were estimated using conditional logistic regression, conditioning on the merged matched risk sets. Both main effects and interaction models were fitted. The interaction model contained the abovementioned exposures, and the interaction term between *metabolic disease* and *IBD* was found statistically significant (Wald *p*-value = 0.0018). The interaction term between *non-IBD autoimmune disease* and *metabolic disease*, and the interaction term between *non-IBD autoimmune disease* and *IBD* were not statistically significant; thus, they were not included in the model. In an additional analysis, the IBD exposure variable was separately categorized into IBD with PSC and IBD without PSC. As a sensitivity analysis, IBD was redefined as having at least two separate IBD diagnoses. The models were refitted and generated comparable results (not shown). To investigate a dose–response relationship of metabolic comorbidity, the hazard of EOCRC was compared between individuals with ≥2, 1, and 0 metabolic diagnoses, excluding individuals with IBD.

Data processing and descriptive tables were performed and created in SAS Version 9.4. All other data analyses were performed in R Version 4.2.0, using the *survival* package (v3.4-0; Therneau, 2022) for conditional logistic regression.

### 2.5. Ethics Statement

This study was approved by the Regional Board of the Ethical Committee in Stockholm, Sweden (DNR: 2014/71–31, 2018/328–32, 2021–00342).

## 3. Results

In total, 2626 patients with EOCRC diagnosis were included in this study, along with 15,756 controls. In both groups, the mean age was 42.5 (SD 5.91) years, and 46.3% were females (Table 1). Education and income levels did not differ statistically significant between the groups. Overall, the prevalence of autoimmune diseases was higher among EOCRC cases (11.1%) compared with controls (6.0%). The observed difference was due to higher prevalence of IBD and endocrine autoimmune diseases. In EOCRC cases, 6.0% had IBD compared with 1.1% in controls. Metabolic disease was more prevalent in EOCRC cases compared with controls (38.6% vs. 26.4%). Hypertension (32.5% vs. 22.1%) and T2DM (7.2% vs. 4.9%) were predominant in both groups.

Table 2 displays the clinicopathological characteristics in EOCRC cases. Overall, tumors were distributed with a predominance of the rectum (38.4%) and left colon (33.6%). An exception was represented by IBD patients, who had more right-sided colon cancers. Cases with previous IBD and no metabolic disease (*n* = 97) were younger at diagnosis (mean age 36.8, SD 7.53 vs. 42.5, SD 5.91 years) compared to cases in general. They also showed lower mean BMI of 24.0 (SD 4.1) compared to IBD patients with metabolic disease (29.0, SD 7.0) and all cases (25.7, SD 4.86). Half of the controls with IBD were men; however, among cases with IBD, more than three-quarters were men.

The conditional logistic regression model revealed statistically significant associations between metabolic and autoimmune diseases and the occurrence of EOCRC. Based on the main effects model, an elevated incidence rate for EOCRC was seen in individuals with IBD (HR 5.98, 95% CI 4.78–7.48), non-IBD autoimmune disease (HR 1.28, 95% CI 1.08–1.52), and metabolic disease (HR 1.82, 95% CI 1.67–1.99) (Table 3). In the interaction model, the HR for EOCRC comparing IBD to non-IBD persons was higher among those without metabolic disease than those with metabolic disease (HR 7.52, 95% CI 5.62–10.06 vs. HR 3.65, 95% CI 2.57–5.19). Based on a limited number of persons, those with both IBD and PSC and metabolic disease had a markedly increased CRC incidence (HR 40.24, 95% CI 9.22–175.63) and even higher without metabolic disease (HR 73.53, 95% CI 9.19–588.38). Duration of IBD, measured as time from first registered IBD diagnosis until date of CRC diagnosis, did not differ between cases with and without concomitant metabolic disease. Presence of one metabolic diagnosis was associated with an increased incidence of EOCRC compared to those with more than one diagnosis (HR for one condition vs. no condition 1.99, 95% CI 1.80–2.20, and HR for ≥2 conditions vs. no condition 1.39, 95% CI 1.20–1.62).

## 4. Discussion

In this large, nested case–control study of 2626 EOCRC cases and their matched controls, we estimated associations between autoimmune and metabolic conditions and EOCRC. As expected, preexisting IBD was associated with a sixfold increased incidence of EOCRC. In addition, preexisting metabolic disease was associated with a twofold increased incidence of EOCRC, whereas non-IBD autoimmune disease was not associated with an increased incidence. We also found that IBD cases with metabolic disease had a less increased incidence of EOCRC in comparison to IBD cases without.

Strengths of this study include the population-based setting with an unselected and high number of EOCRC individuals, together with robust data on comorbid diagnoses and prescription of drugs against metabolic conditions. The data in the NPR are registered prospectively from all hospitals in Sweden, and the use of data from the NPR diminishes the risk of report bias. Furthermore, using national data from the SCRCR, with very high reported validity and coverage, together with a large number of matched controls, ensures that the study is representative of the whole Swedish population [26,29].

Until recently, few studies have focused on the association between IBD and EOCRC [23,34]. IBD is a well-known risk factor for CRC, and the inflammation is thought to drive tumorigenesis in the mucosa of the bowel [17,18]. Patients with IBD have a 50% increased overall risk of CRC, which is even higher in combination with PSC and in young-onset IBD cases [35]. Hence, Swedish national guidelines state that cancer screening with colonoscopy in IBD patients should start 10 years after disease onset. Our study demonstrates that the incidence of CRC in IBD patients is more pronounced in young individuals, perhaps even higher than previously reported. Two reports originating from the same database describe, firstly, a fourfold increased risk for EOCRC among “colitis” patients compared with controls and, secondly, a fourfold increased risk for EOCRC among UC and CD patients, respectively [34,36].

An interesting finding was that EOCRC incidence for IBD patients was less pronounced in combination with metabolic disease. EOCRC cases with IBD and metabolic disease had a 36% higher mean BMI and had a higher mean age at cancer diagnosis than cases with IBD alone. In theory, metabolic disease could be a proxy for mild IBD disease, since IBD cases with severe or long-standing inflammation would be less prone to develop lifestyle-related conditions. In support of this, a study with childhood-onset IBD patients described that very early onset of IBD and extensive colitis causes a highly increased risk of CRC [35]. The duration of IBD did not differ between cases and controls in our material. This estimation was limited since we only included exposures up to 10 years prior to EOCRC diagnosis. Another limitation was that we lacked data regarding IBD severity or medication with biopharmaceuticals. Moreover, our data could not tell whether the CRC tumor was revealed due to surveillance or not. Another interesting finding was that the sex ratio among EOCRC cases with IBD was 3:1 for male dominance, compared to 1:1 in controls. A recent meta-analysis confirmed male sex as an independent risk factor for CRC in IBD cases [37].

In this study, a history of metabolic disease showed an almost twofold increased incidence of EOCRC, which is similar to a previous study that reported a 2.5-fold increased incidence in patients with obesity, hyperlipidemia, or diabetes [34]. Other studies have also shown independently increased incidence of EOCRC in patients with obesity and hyperlipidemia [23,38], although EOCRC incidence in diabetes or hypertension patients has been conflicting [39,40,41]. In our material, hypertension was overrepresented among the included metabolic conditions, strengthening that this is an important risk factor for EOCRC. The study was limited by the observational study setting, and future studies with prospective data collection are needed to verify our results. Information on body measures in controls was not available, and the study lacked primary healthcare data. Hence, we may have missed patients with diet-controlled diabetes or impaired glucose tolerance, and a comprehensive assessment of the metabolic syndrome could not be estimated. On the other hand, none of the included pharmaceuticals can be bought in Sweden without a prescription. In all probability, the vast majority of patients with pharmacologically treated T2DM, hypertension, and hyperlipidemia were, therefore, included. In a dose–response assessment, we analyzed if persons with more than one metabolic condition were more prone to develop EOCRC, but we could not demonstrate such an association. We believe, however, that presence of obesity and obesity-related diseases, rather than the specific diagnoses per see, well before the age of 50, could be seen as surrogate markers for a lifestyle that increases the risk of EOCRC. Therefore, it is arguably suitable to analyze these diagnoses together. Although we lack complete data on obesity, the data on other metabolic conditions are robust and should be taken into consideration when calculating the risk of EOCRC in these individuals. As in all observational studies, there is a potential for residual confounding. The Swedish population comprises mostly Caucasians, and information about race is not registered routinely. Whether these results are generalizable to other populations needs to be confirmed.

Autoimmune diseases represent a wide variation of diseases, which all share the mechanism of a self-reactive adaptive immune response. This causes inflammation which, especially if untreated, can influence the risk of cancer development [14]. Previous studies have mainly shown site-specific cancer risk, such as the connection between IBD and CRC risk, autoimmune thyroid disease and thyroid cancer, and dermatomyositis, as well as an increased risk of several cancer forms [14]. Since autoimmunity has been shown to increase in developed countries, just like EOCRC, we aimed to investigate the connection between autoimmunity and EOCRC risk [16]. Except for IBD and PSC, we found no such relationship. PSC is a strong risk factor not only for cholangiocarcinoma but also for CRC [42]. Although our conditional logistic regression model was limited by relatively few cases with PSC, the point estimate for EOCRC incidence was very high in these individuals, confirming the need for close surveillance for CRC in these patients. The mechanism of CRC development in PSC patients is largely unknown, although a change in bile acid excretion from the liver has been proposed as a clue [43].

The Swedish Board of Health and Welfare recommends CRC screening, including individuals from 60–74 years with biennial testing for fecal occult blood. This has started in all healthcare regions in Sweden and is set to be fully implemented in 2026 [10]. Considering the increasing incidence of EOCRC, voices have been raised to lower the cutoff age to 45 years [44]. Regardless of this, in light of our results, the threshold for a colorectal investigation should be low in clinical practice for young individuals with metabolic conditions.

## 5. Conclusions

This study was conducted to shed light on both known and potentially unknown autoimmune and metabolic risk factors for EOCRC, motivated by the worrying increase in incidence since the 1990s. We conclude that patients with IBD or metabolic comorbidities indisputably have an increased risk of EOCRC. Further studies are needed in young IBD patients to personalize surveillance protocols. In addition, presence of metabolic disease in patients <50 years should be considered in future screening recommendations based on individual risk profiles.

## Figures and Tables

**Table 1:** Descriptive statistics of Swedish early-onset colorectal cancer * patients and controls, matched for birth year, sex, and county, between 2007 and 2016.

Characteristics-	-	Controls		Patients		Total	
		(*n* = 15,756)		(*n* = 2626)		(*n* = 18,382)	
		*n*	(%)	*n*	(%)	*n*	(%)
Age at CRC diagnosis (SCRCR), years (mean (SD))		42.5	(5.9)	42.5	(5.9)	-	-
Sex							
Female		7290	(46.3)	1215	(46.3)	8505	(46.3)
Male		8466	(53.7)	1411	(53.7)	9877	(53.7)
Duration of education							
>12 years		6139	(39.0)	973	(37.1)	7112	(38.7)
10–12 years		7611	(48.3)	1331	(50.7)	8942	(48.6)
<9 years		1855	(11.8)	298	(11.3)	2153	(11.7)
Missing		151	(1.0)	24	(0.9)	175	(9.5)
Income group							
Upper quartile		3827	(24.3)	637	(24.4)	4464	(24.3)
Middle two quartiles		7979	(50.6)	1339	(51.0)	9318	(50.7)
Lower quartile		3863	(24.5)	641	(24.3)	4504	(24.5)
Missing		87	(0.6)	9	(0.3)	96	(0.5)
**Comorbid conditions**							
Metabolic disease		4163	(26.4)	1017	(38.7)	5180	(28.2)
Diabetes mellitus type 2		770	(4.9)	189	(7.2)	959	(5.2)
Primary hypertension		3484	(22.1)	854	(32.5)	4338	(23.6)
Hyperlipidemia		1426	(9.1)	281	(10.7)	1707	(9.3)
Obesity		233	(1.5)	41	(1.6)	274	(1.5)
Fatty liver		<10	-	<10	-	10	(0.1)
Autoimmune disease		956	(6.1)	290	(11.0)	1246	(6.8)
IBD		169	(1.1)	158	(6.0)	327	(1.8)
Crohn’s disease		49	(0.3)	32	(1.2)	81	(0.4)
Ulcerative colitis		61	(0.4)	74	(2.8)	135	(0.7)
IBD-unclassified		59	(0.4)	52	(2.0)	111	(0.6)
PSC		7	-	27	(1.0)	34	(0.2)
Autoimmune diseases **		956	(6.1)	290	(11.0)	1246	(6.8)
Diseases of the blood and blood-forming organs		41	(0.3)	8	(0.3)	49	(0.3)
Endocrine diseases		348	(2.2)	73	(2.8)	421	(2.3)
Diseases of the nervous system		51	(0.3)	9	(0.3)	60	(0.3)
Diseases of the eye		68	(0.4)	12	(0.5)	80	(0.4)
Circulatory disease		<10	-	<10	-	<10	-
Diseases of the digestive system, excluding IBD		28	(0.2)	14	(0.5)	42	(0.2)
Diseases of the skin		151	(1.1)	21	(0.8)	172	(0.9)
Diseases of the musculoskeletal system and connective tissue		178	(0.2)	29	(1.1)	207	(1.1)
Diseases of the genitourinary system		29	(0.2)	7	(0.3)	36	(0.2)

* Age < 50 years at diagnosis. ** Organ-specific autoimmune and chronic inflammatory disorders according to the headlines of ICD10. Abbreviations: CRC, colorectal cancer; SCRCR, Swedish Colorectal Cancer Registry; SD, standard deviation; ICD10, International Classification of Disease, 10th edition; IBD, inflammatory bowel disease; PSC, primary sclerosing cholangitis.

**Table 2:** Patient characteristics of Swedish early-onset colorectal cancer* (2007–2016), separated for concomitant comorbid conditions.

	No Comorbid Conditions (*n* = 1465) *n* (%)	Autoimmune Disease without IBD or Metabolic (*n* = 47) *n* (%)	Metabolic Disease without IBD (*n* = 956) *n* (%)	IBD without Metabolic Disease (*n* = 97) *n* (%)	IBD with Metabolic Disease (*n* = 61) *n* (%)	Total (*n* = 2626) *n* (%)
**Age**						
Median, years [min, max]	43.0 (19.0, 49.0)	44.0 (27.0, 49.0)	45.0 (21.0, 49.0)	38.0 (21.0, 49.0)	42.0 (30.0, 49.0)	44.0 (19.0, 49.0)
**Weight**						
Mean, kg (SD)	75.1 (16.1)	79.3 (17.2)	81.9 (18.3)	74.4 (14.6)	91.3 (22.0)	77.9 (17.5)
Missing	275 (18.8)	9 (19.1)	221 (23.1)	14 (14.4)	8 (13.1)	527 (20.1)
**Height**						
Mean, cm (SD)	174 (9.8)	175 (11.6)	174 (9.8)	176 (7.4)	177 (8.2)	174 (9.7)
Missing	304 (20.8)	9 (19.1)	243 (25.4)	17 (17.5)	9 (14.8)	582 (22.2)
**BMI**						
Mean, kg/m^2^ (SD)	24.8 (4.3)	25.9 (4.5)	27.1 (5.2)	24.0 (4.1)	29.0 (7.0)	25.7 (4.9)
Missing	309 (21.1)	9 (19.1)	245 (25.6)	17 (17.5)	9 (14.8)	589 (22.4)
**Sex**						
Male	763 (52.1)	21 (44.7)	508 (53.1)	73 (75.3)	46 (75.4)	1411 (53.7)
Female	702 (47.9)	26 (55.3)	448 (46.9)	24 (24.7)	15 (24.6)	1215 (46.3)
**Duration** **of education**						
<9 years	150 (10.2)	2 (4.3)	126 (13.2)	14 (14.4)	6 (9.8)	298 (11.3)
10–12 years	717 (48.9)	26 (55.3)	512 (53.6)	46 (47.4)	30 (49.2)	1331 (50.7)
12> years	589 (40.2)	19 (40.4)	304 (31.8)	36 (37.1)	25 (41.0)	973 (37.1)
Missing	9 (0.6)	-	14 (1.5)	1 (1.0)	-	24 (0.9)
**Income group**						
Lower quartile	352 (24.0)	13 (27.7)	223 (23.3)	35 (36.1)	18 (29.5)	641 (24.4)
Middle two quartiles	744 (50.8)	21 (44.7)	498 (52.1)	41 (42.3)	35 (57.4)	1339 (51.0)
Upper quartile	362 (24.7)	13 (27.7)	234 (24.5)	21 (21.6)	7 (11.5)	637 (24.3)
Missing	7 (0.5)	-	1 (0.1)	-	1 (1.6)	9 (0.3)
**ASA Class**						
1	729 (49.8)	13 (27.7)	314 (32.8)	21 (21.6)	9 (14.8)	1086 (41.4)
2	443 (30.2)	22 (46.8)	371 (38.8)	56 (57.7)	28 (45.9)	920 (35.0)
3	71 (4.8)	4 (8.5)	95 (9.9)	7 (7.2)	16 (26.2)	193 (7.3)
4	8 (0.5)	-	<5	<5	-	12 (0.5)
Missing	214 (14.6)	8 (17.0)	173 (18.1)	12 (12.4)	8 (13.1)	415 (15.8)
**Cancer location**						
Right-side colon cancer	402 (27.4)	14 (29.8)	251 (26.3)	37 (38.1)	31 (50.8)	735 (28.0)
Left-side colon cancer	490 (33.4)	18 (38.3)	327 (34.2)	28 (28.9)	17 (27.9)	880 (33.5)
Rectal cancer	569 (38.8)	15 (31.9)	372 (38.9)	32 (33.0)	12 (19.7)	1000 (38.1)
Missing	4 (0.3)	-	6 (0.6)	-	1 (1.6)	11 (0.4)
**Tumor stage**						
I	220 (15.0)	9 (19.1)	117 (12.3)	17 (17.5)	10 (16.4)	373 (14.2)
II	334 (22.8)	13 (27.7)	170 (17.8)	15 (15.5)	17 (27.9)	549 (20.9)
III	442 (30.2)	12 (25.5)	273 (28.6)	36 (37.1)	19 (31.1)	782 (29.8)
IV	428 (29.2)	10 (21.3)	360 (37.7)	25 (25.8)	12 (19.7)	835 (31.8)
Missing	41 (2.8)	3 (6.4)	36 (3.8)	4 (4.1)	3 (4.9)	87 (3.3)
**Metabolic disease**						
Type 2 diabetes	-	-	178 (18.6)	-	11 (18.0)	189 (7.2)
Hypertension	-	-	807 (84.4)	-	47 (77.0)	854 (32.5)
Lipidemia	-	-	254 (26.6)	-	27 (44.3)	281 (10.7)
Obesity	-	-	35 (3.7)	-	6 (9.8)	11 (0.4)
Fatty liver	-	-	<5	-	<5	<5
Two or more of the above	-	-	238 (24.9)	-	19 (31.1)	257 (9.8)
**IBD condition**						
Ulcerative colitis	-	-	-	46 (47.4)	28 (45.9)	74 (2.8)
Crohn’s disease	-	-	-	19 (19.6)	13 (21.3)	32 (1.2)
IBD-unclassified	-	-	-	32 (33.0)	20 (32.8)	52 (2.0)

* Age < 50 years at diagnosis. Abbreviations: CRC, colorectal cancer; SD, standard deviation; BMI, body mass index; IBD, inflammatory bowel disease; ASA, American Society of Anesthesiologists.

**Table 3:** Conditional logistic regression models of the incidence of early-onset colorectal cancer conditioning on matched risk set, with covariates: indicators for non-IBD autoimmune disease, metabolic disease, IBD, and an interaction term between metabolic disease and IBD.

Comparison	Cases (Yes/No)	Controls (Yes/No)	Main Effect Model HR (95% CI)	Interaction Model HR (95% CI)
Non-IBD autoimmune disease	172/2454	816/14,940	1.28 (1.08–1.52)	0.97 (0.81–1.16)
Metabolic disease	1017/1609	4163/11,593	1.82 (1.67–1.99)	-
without IBD	956/1512	4092/11,495	-	1.82 (1.66–2.00)
IBD	158/2468	169/15,587	5.98 (4.78–7.48)	-
with metabolic disease	61	71	-	3.65 (2.57–5.19)
without metabolic disease	97	98	-	7.52 (5.62–10.06)

Abbreviations: IBD, inflammatory bowel disease; HR, hazard ratio; CI, confidence interval.

## Data Availability

The CRCBaSe data are stored securely on a guest server at Karolinska Institutet, Stockholm and can be provided upon request.

## Supplementary Materials

The following supporting information can be downloaded at https://www.mdpi.com/article/10.3390/cancers15030688/s1: Table S1. Diagnoses and their respective ICD-10 codes used to identify comorbid conditions in cases and controls; Table S2. Source of identified metabolic comorbidities in early-onset colorectal cancer patients, diagnosed in 2007–2016, and controls.
