# Supplementary material for: Autoimmune and Metabolic Diseases and the Risk of Early-Onset Colorectal Cancer, a Nationwide Nested Case–Control Study
Source: Cancers (Basel). 2023 Jan 22;15(3):688. doi: 10.3390/cancers15030688 (PMC9913656; doi:10.3390/cancers15030688)
Supplement: Supplementary file 1 [file cancers-15-00688-s001.zip › cancers-2120345-supplementary.pdf]

Table S1. Diagnoses and their respective ICD-10-codes used to identify comorbid conditions in cases and controls.

Diagnoses	ICD-10-codes
**Metabolic conditions**	
Type 2 diabetes mellitus	E11
Essential (primary) hypertension	I10
Disorders of lipoprotein metabolism and other lipidemias	E78
Overweight and obesity	E66
Fatty (change of) liver, not elsewhere classified	K76.0
**Inflammatory bowel disease and primary sclerosing cholangitis**	
Crohn’s disease	K50
Ulcerative colitis	K51
Indeterminate colitis	K52.3, K52.9
Primary sclerosing cholangitis	K83.0
**Autoimmune and chronic inflammatory conditions**	
**Diseases of the blood and blood-forming organs and certain disorders involving the immune mechanism.**	
Vitamin B12 deficiency anemia due to intrinsic factor deficiency	D510
Other autoimmune hemolytic anemias	D59.1
Other thrombophilia	D68.6
Immune thrombocytopenic purpura	D69.3
Sarcoidosis	D86
**Endocrine diseases**	
Hypothyroidism, other nontoxic goiter, thyrotoxicosis, thyroiditis	E03-E06, excl. E06.0, E06.4
Type 1 diabetes mellitus	E10
Addison, Primary adrenocortical insufficiency	E27.1
Autoimmune polyglandular failure	E31.0
Non-neuropathic heredofamilial amyloidosis	E85.0
**Diseases of the nervous system**	
Multiple sclerosis	G35
Inflammatory polyneuropathy, Guillain Barré	G61
Myasthenia gravis	G70.0
**Diseases of the eye and adnexa**	
Scleritis	H15
Iridocyclitis	H20, excl. H20.2
**Diseases of the circulatory system**	
Rheumatic fever, other rheumatic heart diseases	I00-I09
Diseases of the digestive system excl. IBD	
Primary biliary cholangitis	K74.3
Autoimmune hepatitis	K75.4
Intestinal malabsorption	K90.0
Diseases of the skin	
Dermatitis (pemphigoid)	L10.0, L12
Psoriasis	L40
Erythema nodosum	L52
Vitiligo	L80
Pyoderma gangrenosum	L88
**Diseases of the musculoskeletal system and connective tissue**	
Reactive arthropathies	M02
Post infective arthropathies	M03
Rheumatoid arthritis	M05-M06
Psoriatic arthropathy	M07.2–3
Juvenile arthritis	M08
Juvenile arthritis in psoriasis	M09.0
Arthritis	M13
Polyarteritis nodosa and related conditions	M30

Other necrotizing vasculopathies	M31
Systemic lupus erythematosus	M32
Dermatomyositis	M33
Systemic sclerosis	M34
Other systemic involvement of connective tissue	M35, excl. M35.7
Ankylosing spondylitis	M45
Other inflammatory spondylopathies	M46.0–1;8–9
Rheumatism, unspecified	M79.0
Relapsing polychondritis	M94.1
Arteritis, unspecified	I77.6
**Diseases of the genitourinary system**	
Acute nephritic syndrome	N00
Rapidly progressive nephritic syndrome	N01
Recurrent and persistent hematuria	N02
Chronic nephritic syndrome	N03
Nephrotic syndrome	N04
Unspecified nephritic syndrome	N05

Table S2. Source of identified metabolic comorbidities in Early onset colorectal cancer patients, diagnosed 2007-2016, and controls.

Diagnose (ICD-code)	Controls, n source			Cases, n Source		
	**IPR**	**PDR**	**OPR**	**IPR**	**PDR**	**OPR**
Type 2 diabetes mellitus (E11)*	80	753	108	21	187	35
Disorders of lipoprotein metabolism and other lipidemias (E78)**	49	1409	62	15	276	10
Primary hypertension (I10)***	137	3467	154	43	848	39

Abbreviations: ICD, international classification of disease, IPR, In-Patient Registry, PDR, Prescribed Drugs Registry, OPR, Out-Patient Registry. *ATC-codes: A10, Insulins and analogues for injection, Blood glucose lowering drugs, excl. insulins. **ATC-codes: C03, C07-C09, Diuretics, beta blocking agents, agents acting on the renin-angiotensin system. ***ATC-codes: C10, Lipid modifying agents.
